# Pneumatosis Intestinalis and Concurrent Focal Diaphragmatic Pneumatosis Cystoides Presenting With Pneumoperitoneum in a 20 Year‐Old Male: A Case Report

**DOI:** 10.1002/ccr3.70063

**Published:** 2025-01-02

**Authors:** Manal Fasih, Patricia Colucci, Kara Monday

**Affiliations:** ^1^ Burnett School of Medicine at Texas Christian University Fort Worth Texas USA; ^2^ Baylor University Medical Center Dallas Texas USA; ^3^ Baylor Scott & White All Saints Medical Center Fort Worth Texas USA

**Keywords:** case reports, intestinal malrotation, pneumatosis cystoides intestinalis, pneumoperitoneum

## Abstract

Pneumatosis cystoides intestinalis can present with concurrent diaphragmatic cysts, a previously undocumented phenomenon. Surgical management may be required, but further investigation is needed to understand the pathogenesis and optimize management in atypical and chronic cases, such as this case with a history of corrected intestinal malrotation.

## 1 Introduction

Pneumoperitoneum, defined as air within the peritoneal cavity, is typically a sign of a perforation of an abdominal viscus for which emergent surgical management is indicated [1]. A rare cause of pneumoperitoneum is pneumatosis cystoides intestinalis, which is characterized by subserosal or submucosal cysts of the gastrointestinal (GI) tract [2]. Potential sources of gas that could lead to pneumatosis intestinalis (PI) include intraluminal gas, bacterial gas, and pulmonary gas [3, 4, 5]. Previously, about 85% of PI cases have been found to be associated with a known condition, such as mesenteric or intestinal ischemia [6], infectious etiology, collagen vascular disease (e.g., scleroderma), iatrogenic etiology (e.g., due to endoscopic procedure), medications (e.g., corticosteroids, lactulose, chemotherapeutic agents), autoimmune disease [7, 8], and pulmonary diseases (e.g., chronic obstructive pulmonary disease and asthma) [9], with the remaining being idiopathic [10]. Surgical intervention is typically reserved for severe cases of PI (e.g., complicated by bowel necrosis, obstruction, or involvement of pneumoperitoneum indicative of bowel perforation) [11].

To our knowledge, no cases of PI with concurrent focal pneumatosis cystoides occurring from outside of the GI tract have been documented. Further investigation into varying presentations and pathogenesis and their associated outcomes of surgical versus nonoperative treatment are warranted to better characterize this disease. This is a report of a 20‐year‐old male with a history of congenital malrotation who presented with pneumoperitoneum secondary to PI of unknown etiology, and focal diaphragmatic pneumatosis cystoides of unknown significance who underwent surgical management.

## 2 Case History/Examination

A 20 year‐old Caucasian male with a past medical history of lactose intolerance and COVID‐19 presented with over a year of ongoing abdominal pain and distention. The patient had military training history but no contributing travel or family history. In the fall of 2021, the patient underwent an exploratory laparoscopy after presenting with abdominal distension and a computerized tomography (CT) scan suggestive of pneumoperitoneum and free abdominal fluid. During this first exploratory laparoscopy, congenital malrotation was identified as the cecum was in the right upper quadrant and was corrected by Ladd's procedure with adhesiolysis. During surgery, the mid‐section of the ileum appeared inflamed. At that juncture, there was no clear etiology for the free air in the peritoneum. Ascitic fluid cell analysis and peritoneal lymph node biopsy were negative for malignancy. Lymph node biopsy showed mesothelial‐lined fibrovascular tissue with chronic reactive and inflammatory changes. He was discharged after tolerating an advancing diet.

In the winter of 2021, he noticed a return of intermittent abdominal bloating and discomfort, decreased appetite, along with generalized weakness and weight loss. He reported intermittent distention, where for 24–48 h, he would experience abdominal distension with spontaneous resolution without passage of gas. He then underwent colonoscopy and esophagogastroduodenoscopy (EGD), which were unremarkable.

His chronic symptoms of mild abdominal pain and distention acutely worsened 2–3 weeks before presenting to our emergency department (ED) in the fall of 2022. His lower abdominal pain worsened and spread to the right upper quadrant without relief from simethicone or dicycloverine. Upon presenting to ED, his vital signs were stable and moderate abdominal distension was appreciated on physical exam. There were no focal peritoneal signs.

## 3 Methods (Differential Diagnosis, Investigations, and Treatment)

**FIGURE 1:**
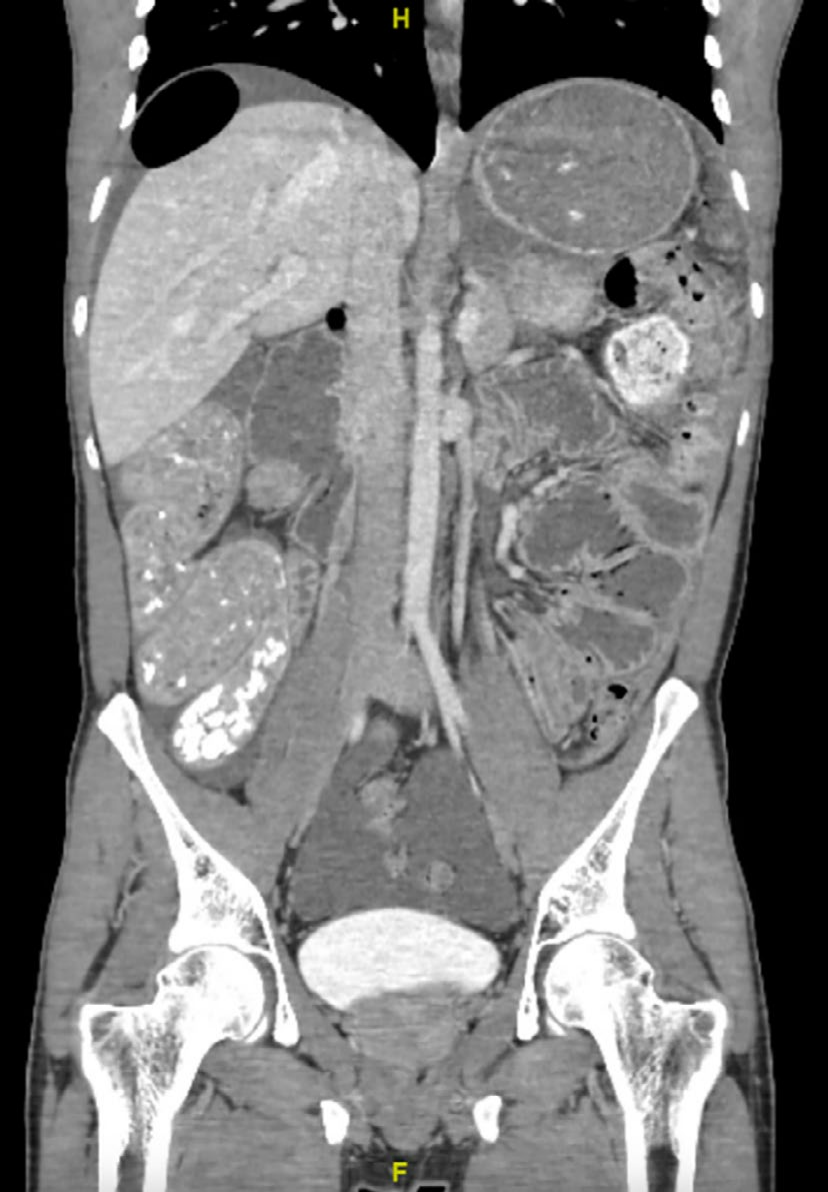
Coronal cross‐section of CT abdomen and pelvis with diffuse fluid‐filled loops of bowel and a focal section of air underneath the diaphragm in the right upper quadrant. H: toward patient head and F: toward patient feet.

**FIGURE 2:**
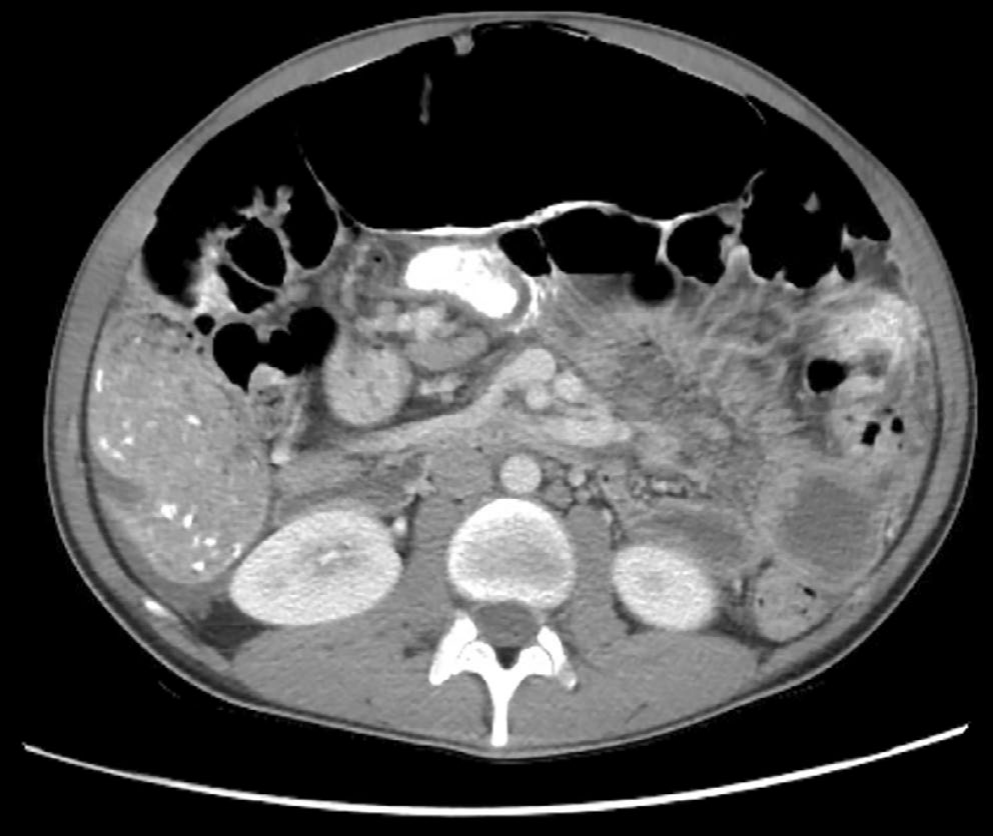
Transverse cross‐section of abdominal CT without contrast revealing distended loops of bowel.

**FIGURE 3:**
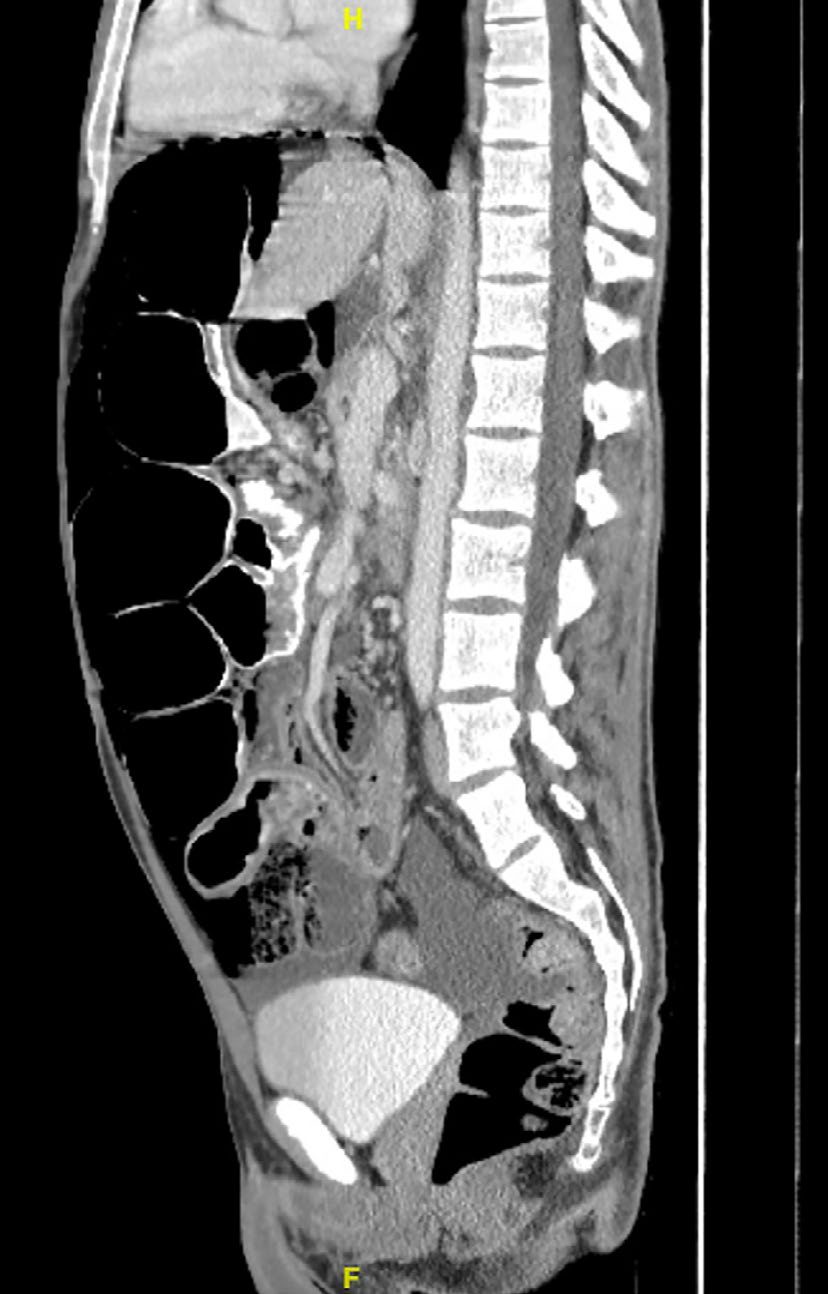
Sagittal cross‐section of CT abdomen and pelvis revealing distended loops of bowel. H: toward patient head and F: toward patient feet.

Laboratory values including white blood cell count, renal function and lactate were within normal limits. CT scan of the abdomen and pelvis showed pneumoperitoneum with a large amount of free air in the upper abdomen, with associated mild ascites (Figure 1). No clear location of ruptured viscus or inflammation was visible. There were also fluid‐filled distended loops of small bowel and massive distention of the mid‐transverse colon (Figure 2) and right colon (Figure 3) containing air, stool, and opaque material.

**FIGURE 4:**
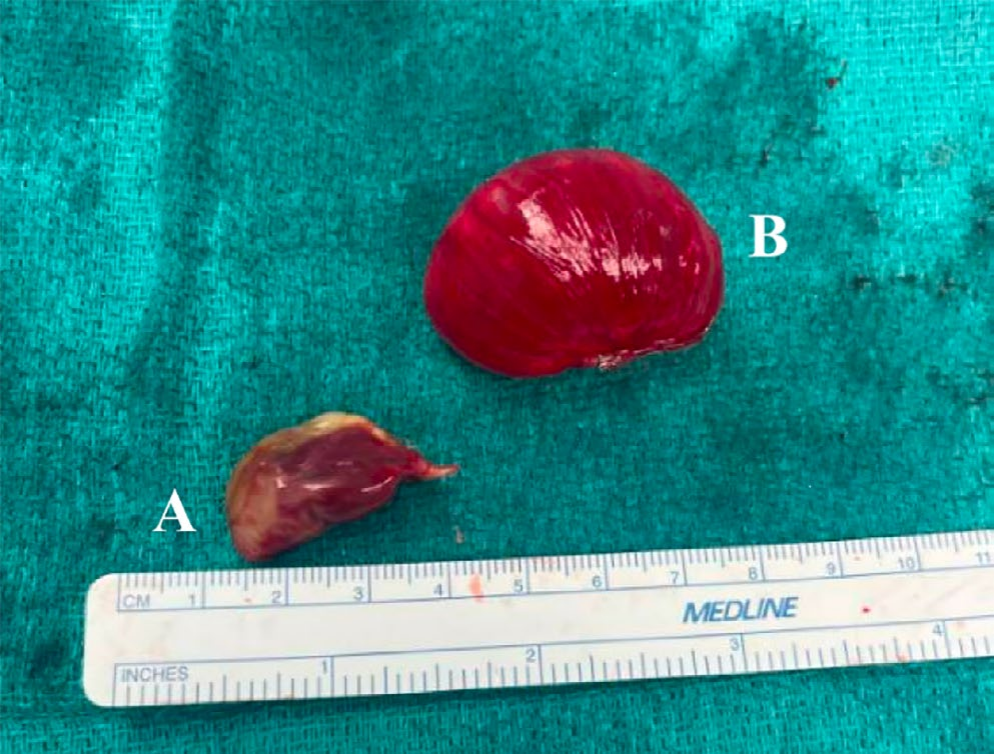
Gross examination of two masses, specimen A (left) and B (right) resected from the diaphragm.

**FIGURE 5:**
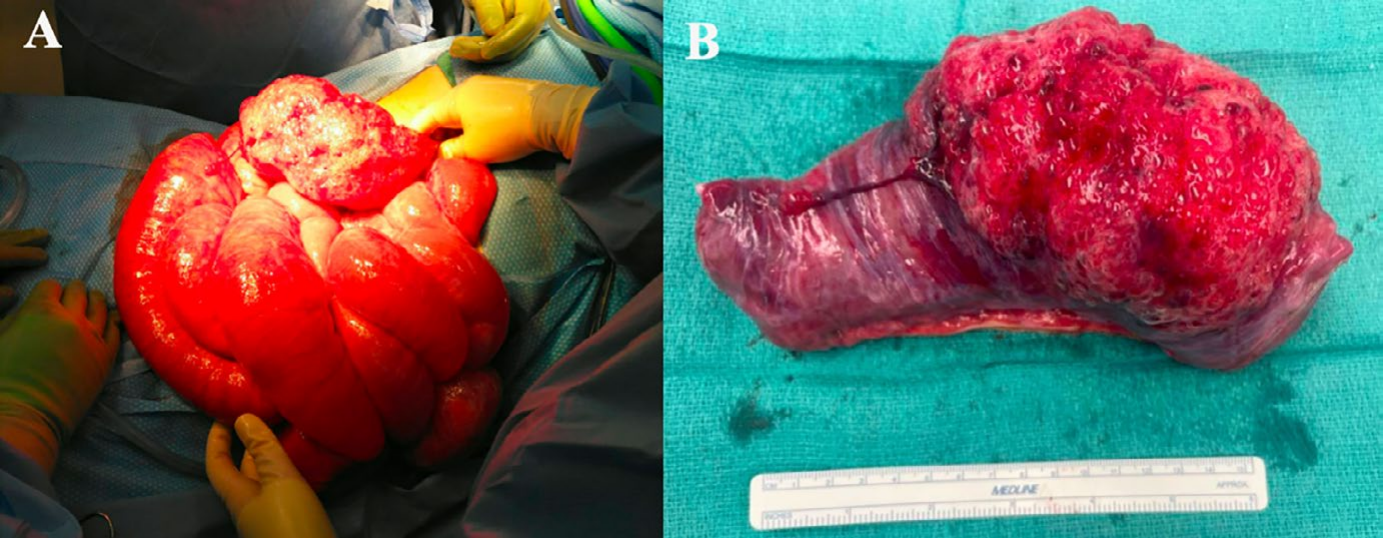
Gross specimen of dilated loops of bowel (B) including PI examined in the operating field (A).

The patient underwent urgent diagnostic laparoscopy. On initial inspection, the bowel was distended, but was pink and well‐perfused, with white, non‐exudative streaking in the wall of the bowel. Several liters of thin turbid brown‐tinged/green fluid were visualized in the abdomen. Because of challenging visualization secondary to significant bowel distension, the surgery was converted to open. The right colon was adherent to the right abdominal wall at the appendectomy site causing some partial malrotation. The adhesions/Ladd's bands were lysed, freeing the entire bowel. Over the dome of the liver was a round, thin‐walled air‐filled structure, off a pedicle to the diaphragm (Figure 4). This was stapled and ligated off the pedicle. There were also cystic/pneumatosis‐like changes over the diaphragm and anterior peritoneal covering of the abdominal wall (Figure 4). There was an area of exuberant pneumatosis of small bowel in the left upper quadrant, but no distinct perforation was visualized (Figure 5). This was determined to be the most likely source of pneumoperitoneum, so a stapled bowel resection was done, and two drains were left in place. Stapled small bowel anastomosis was performed rather than hand‐sewn because of evidence showing reduced operative time with stapled small bowel anastomosis without a significant increase in complications (e.g., anastomotic leak) [12, 13, 14, 15].

## 4 Conclusions and Results (Outcome and Follow‐Up)

Pathologic findings of the specimens are summarized below:

- Diaphragmatic masses (Figure 4):
  ○ Specimen A (Figure 4A): 2.5 × 1.7 × 1.1 cm of partially cystic necrotic tissue surrounded by a thin rim of viable hyalinized fibrocollagenous tissue, consistent with infarct.
  ○ Specimen B (Figure 4B): 3.8 × 2.7 × 2.5 cm cyst with a rim of moderately cellular fibrocollagenous tissue, consistent with focal pneumatosis cystoides.
- Bowel resection (Figure 5B):
  ○ Fragments of terminal ileum, margins unremarkable, no evidence of necrosis, minimal loss of villi.
  ○ Grossly spongy areas: composed of complex interlacing cysts lined by polygonal histiocytes, occasionally pseudostratified.
    - Cystic change was largely subserosal but also involved the muscularis propria and mucosa.
  ○ Stroma: composed of modestly cellular myxoid fibrocollagenous tissue with admixed round inflammatory cells.
  ○ Central serosal surface contained a multicystic mass (14.0 × 10.0 × 1.5 cm), with subcentimeter watery‐fluid containing cysts.
    - Thick, turbid, yellow, purulent material was revealed at the antimesenteric aspect.
  ○ Final pathologic diagnosis: segmental pneumatosis cystoides intestinalis.
- Peritoneal fluid analysis:
  ○ Abundant acute and chronic inflammation in a background of reactive mesothelial cells and histiocytes.
  ○ Negative for infection or malignancy.

The patient remained stable postoperatively. On postoperative Days 1–3 he was on a nothing‐by‐mouth (NPO) diet, had bilious nasogastric (NG) tube output, and required ketamine for pain control. He started a 4‐day course of piperacillin/tazobactam and fluconazole. By postoperative Day 4, his pain improved, a clear liquid diet was started, and the NG tube was removed. He was discharged on postoperative Day 5.

**FIGURE 6:**
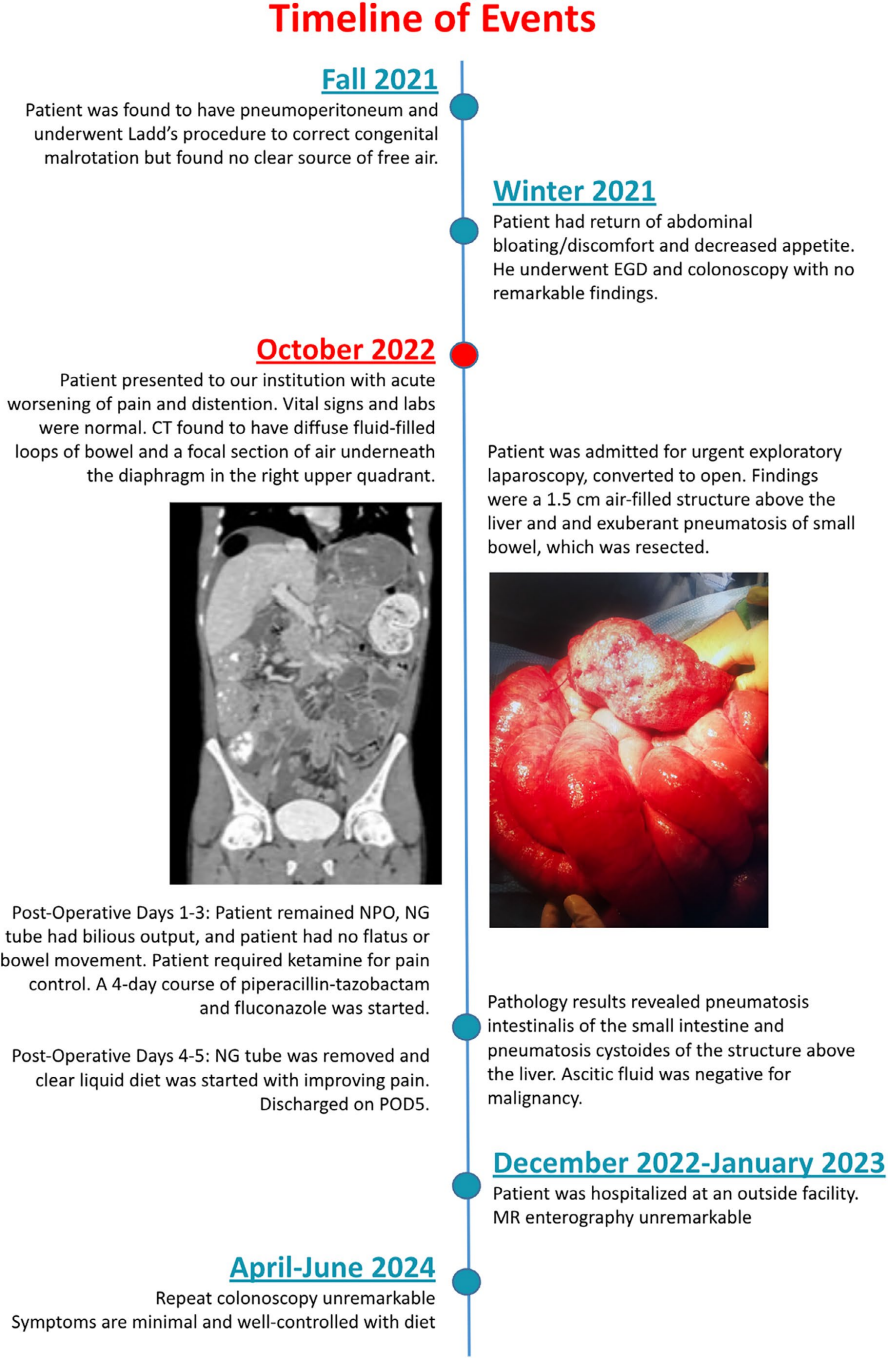
Timeline of events in this case presentation.

Several weeks after discharge, the patient was hospitalized at another facility for continued symptoms. The patient reported undergoing a magnetic resonance (MR) enterography at that time which was unremarkable. Additionally, the patient reported undergoing a repeat colonoscopy in April of 2024 with no significant findings. In June of 2024, the patient reported that, symptomatically, he had minimal acid reflux, bloating, and abdominal distention/pain that were well‐controlled by limiting caloric intake. His weight remained stable despite limiting his diet. A chronological timeline of events of this case is outlined in Figure 6.

## 5 Discussion

This case highlights a unique presentation of pneumoperitoneum secondary to PI with concurrent focal pneumatosis cystoides. The concurrent presence of pneumatosis cystoides occurring from outside of the GI tract, the diaphragm in this case, has not been documented in the literature. The patient's clinical course was complicated by recurrent abdominal symptoms and multiple interventions. Despite initial surgical management of the malrotation, the patient continued to experience symptoms, necessitating further investigation and repeat procedures. The persistence of symptoms and the discovery of diaphragmatic pneumatosis cystoides suggest that the initial surgery may not have fully addressed the underlying pathophysiology. Alternatively, the waxing and waning nature of symptoms could be indicative of chronic PI. Surgical intervention remains the mainstay of treatment for PI when complications such as bowel obstruction or perforation are present [11]. In this case, the decision to perform a bowel resection and resect the diaphragmatic cysts was guided by the acute nature of the presentation, despite the lack of clear perforation. The patient's postoperative recovery was complicated by persistent symptoms.

The etiology of PI remains idiopathic in many cases [10]. Our patient had no apparent underlying cause of the PI, as autoimmune disease (e.g., Crohn's disease) and causes of barotrauma are unlikely, despite history of endoscopy and colonoscopy procedures prior to presentation. Notably, the patient had a history of Coronavirus disease 2019 (COVID‐19) prior to the clinical course, raising the question of a potential association between viral infection and PI [10]. Other potential infectious associations with PI include cytomegalovirus in post‐lung transplant patients [16], cryptosporidiosis, human immunodeficiency virus (HIV) [17], *Clostridium difficile* , rotavirus, and adenovirus in children [18]. An infectious cause is not likely in this patient because of his normal white blood cell count and lack of infectious signs in the pathologic studies. Additionally, the patient was not immunocompromised and did not have any risk factors for an opportunistic infection.

The significance of the diaphragmatic pneumatosis cystoides is uncertain. Abnormalities of the diaphragm were not specifically noted during his initial surgery, thus the time of onset of this cyst is unknown and could theoretically be sequelae of the pneumatosis cystoides intestinalis, which is unlikely given the negative margins of the PI. The occurrence of pneumatosis cystoides in two separate locations, the diaphragm and intestine, suggests a possible systemic/tissue predisposition to cyst formation. Collagen vascular disorders were considered but are unlikely because of the absence of characteristic clinical signs. This presentation raises the possibility of an unidentified connective tissue disorder or a rare presentation of an existing one.

An important consideration in this case is the patient's history of congenital malrotation, which was surgically corrected prior to presenting with PI. Although malrotation is most commonly diagnosed in early childhood with other concurrent anomalies, many cases have also been reported in adolescents and adults [19]. PI is not a commonly reported complication after Ladd's procedure in children [20], however limited research has investigated long‐term outcomes of the Ladd's procedure performed in adults.

This case highlights the need for further investigation into the pathogenesis and optimal management strategies for PI, particularly in atypical presentations, to improve patient outcomes and expand our understanding of this rare condition. Additionally, future studies should evaluate the long‐term outcomes of Ladd's procedure when performed in adults. Genetic and pathology studies may uncover underlying connective tissue disorders predisposing patients to cyst formation, particularly in the context of congenital malrotation. Long‐term follow‐up of patients with PI is essential to monitor for recurrence and manage chronic symptoms.

## Author Contributions

**Manal Fasih:** conceptualization, data curation, investigation, visualization, writing – original draft, writing – review and editing. **Patricia Colucci:** conceptualization, data curation, investigation, visualization, writing – review and editing. **Kara Monday:** conceptualization, data curation, investigation, supervision, writing – review and editing.

## Ethics Statement

The authors have nothing to report.

## Consent

Written informed consent was obtained from the patient for publication of this case report and any accompanying images.

## Conflicts of Interest

The authors declare no conflicts of interest.

## Data Availability

The authors have nothing to report.
